# Relationship between TyG-related index and hearing loss in people over 45 s in China

**DOI:** 10.3389/fpubh.2025.1506368

**Published:** 2025-02-05

**Authors:** Chao Wang, Mengdi Shi, Liangzhen Xie, Chenhao Jiang, Yunxin Li, Jingxiao Li, Shulin Li, Yan Li

**Affiliations:** ^1^Heilongjiang University of Traditional Chinese Medicine, Harbin, China; ^2^The First Affiliated Hospital of Heilongjiang University of Traditional Chinese Medicine, Harbin, China; ^3^Damao United Banner Mongolian Medical Hospital, Baotou, China; ^4^The Second Affiliated Hospital of Heilongjiang University of Traditional Chinese Medicine, Harbin, China

**Keywords:** BMI, TyG-BMI index, aging-related, CHARLS, hearing loss

## Abstract

**Background:**

Triglycerides and fasting glycemic index are biomarkers used to assess the risk of insulin resistance and metabolic syndrome. It and its derivatives such as the TyG-BMI index are currently able to reflect the degree of insulin resistance and are closely related to a variety of metabolic diseases. The relationship between the TyG-BMI index and hearing loss remains underexplored, particularly in the context of aging and public health. This study investigates the association of the TyG index, BMI index, and TyG-BMI index with hearing loss, with a focus on their potential implications for the aging population.

**Methods:**

Data from the China Health and Retirement Longitudinal Study (CHARLS) database were analyzed using R software. We applied multi-factor logistic regression, linear regression, restricted cubic splines, and subgroup analyses to assess the impact of the TyG index, BMI index, and TyG-BMI index on hearing loss across different age groups.

**Results:**

The TyG index was not significantly associated with hearing loss. However, both the BMI index and the TyG-BMI index exhibited a positive correlation with hearing loss, particularly among older individuals. The results suggest that as the population ages, higher BMI and TyG-BMI indices may increase the risk of hearing impairment.

**Conclusion:**

While the TyG index does not show a significant link to hearing loss, higher BMI and TyG-BMI indices are associated with an increased risk of hearing loss, especially in older adults. These findings highlight the importance of considering aging-related factors in public health initiatives aimed at preventing hearing loss. Further research is needed to elucidate the mechanisms underlying these associations and to develop age-inclusive strategies for addressing hearing impairment in the aging population.

## Background

Hearing loss is a prevalent condition that significantly impacts individuals’ quality of life and functional capabilities (1–3). As the global population ages, understanding the risk factors contributing to hearing impairment becomes increasingly important. Among the various factors influencing hearing loss, metabolic disorders and obesity have garnered considerable attention (4–6). Recent studies have begun to investigate how metabolic indices and anthropometric measures relate to hearing loss, with a particular focus on the TyG (triglyceride-glucose) index and body mass index (BMI) (7, 8).

The TyG index, a surrogate marker for insulin resistance, combines fasting triglyceride levels and fasting glucose levels to provide insights into metabolic health. It has been associated with various health outcomes, including cardiovascular disease and diabetes (9–11). The relationship between the TyG index and hearing loss, however, remains less explored. The BMI, a well-established indicator of general obesity, is also implicated in various health conditions, but its direct link to hearing loss requires further examination (12). Obesity has been suggested to contribute to hearing impairment through systemic inflammation, oxidative stress, and altered blood flow, but the extent of this relationship and its underlying mechanisms are not fully understood (13, 14). The TyG-BMI index, an integrative measure combining both the TyG and BMI indices, represents a novel approach to exploring the multifaceted interactions between metabolic and obesity-related factors in relation to hearing loss. The TyG-BMI index offers several advantages over examining individual indices alone.

First, the TyG-BMI index combines two important health markers—metabolic dysfunction (represented by the TyG index) and general obesity (represented by BMI)—into a single composite measure. This integration allows for a more comprehensive evaluation of how combined metabolic and obesity-related factors influence auditory health. By capturing both dimensions of metabolic and physical health, the TyG-BMI index can provide a more nuanced understanding of their joint effects on hearing impairment.

Second, using the TyG-BMI index can improve the precision of risk assessments for hearing loss. While the TyG index and BMI individually offer insights into different aspects of health, their combined effect might better capture complex interactions that contribute to hearing impairment. This could enhance the accuracy of identifying individuals at higher risk of hearing loss and help in tailoring more effective preventive and therapeutic strategies.

Third, the TyG-BMI index may reveal potential synergistic effects that are not apparent when examining each index separately. For example, an individual with both high TyG and high BMI might face compounded risks of hearing loss that are greater than the sum of risks posed by each factor alone. Identifying such synergistic effects can lead to better-targeted interventions and a deeper understanding of the pathophysiology underlying hearing impairment (15–19).

This study leverages data from the CHARLS (China Health and Retirement Longitudinal Study) database, a comprehensive dataset that provides a rich source of information on various health indicators and outcomes. Using R software, advanced statistical techniques were employed to analyze the data. Multi-factor logistic and linear regression models were applied to assess the relationships between the TyG index, BMI, TyG-BMI index, and hearing loss. Additionally, restricted cubic splines were utilized to explore potential non-linear associations, and subgroup analyses were conducted to determine whether the relationships varied across different populations.

Understanding these relationships is crucial for developing targeted interventions and preventive measures to mitigate hearing loss. This study aims to provide valuable insights into how metabolic and obesity-related indices are linked to auditory health, potentially guiding future research and informing clinical practices. As the evidence base grows, it will be essential to continue exploring these associations to better understand the complex interactions between metabolic health, obesity, and hearing impairment.

## Methods

### Data source

This study utilized data from the CHARLS, a nationally representative longitudinal survey designed to collect comprehensive information on the health, economic status, and social characteristics of older adults in China (20). CHARLS provides a rich dataset that includes variables related to health indicators, lifestyle factors, and hearing assessments. The analysis focused on evaluating the relationships between the TyG index, BMI, the TyG-BMI index, and hearing loss.

### Study population

**Figure 1:**
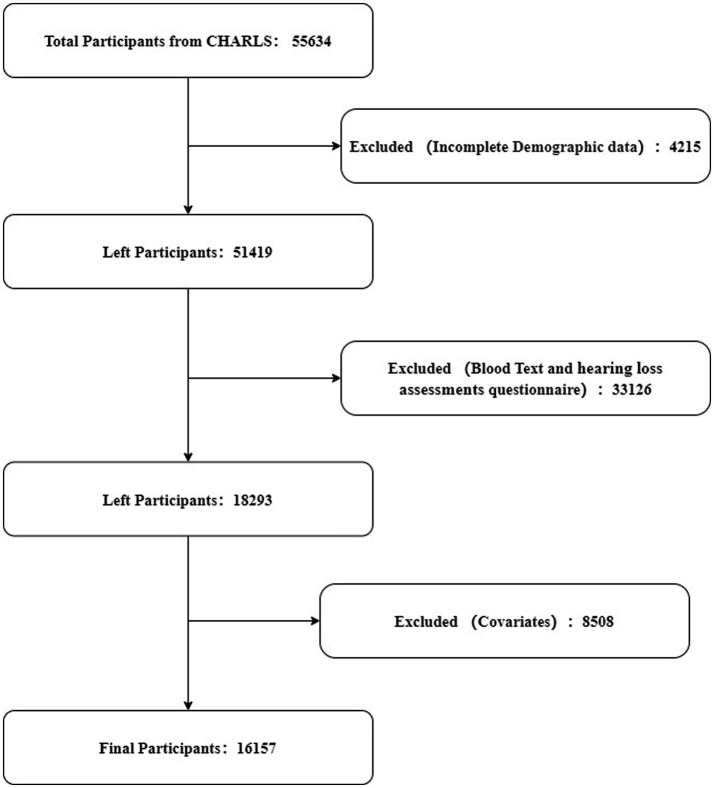
Flow chart of the study population.

The study included participants aged 45 years and older from the CHARLS database. Inclusion criteria required complete data on fasting glucose, triglyceride levels, BMI, and hearing loss assessments. Participants with incomplete or missing data in these areas were excluded. From an initial pool of 55,634 participants, 4,215 were removed due to incomplete demographic information. Subsequently, 33,126 were excluded for not fully completing both hearing loss assessments and blood tests. Additionally, 8,508 participants were excluded due to missing covariate data. Consequently, 16,157 participants were included in the final analysis, as depicted in Figure 1.

### Variables and measures

Hearing loss was assessed using a standardized questionnaire from CHARLS. Participants reported their ability to hear without difficulty, with responses categorized as “hearing loss” or “no hearing loss” (21).

### Triglyceride glucose index, BMI and TyG-BMI index

TyG Index: The TyG index was computed by using the following formula: Ln (fasting triglycerides [mg/dl] *fasting blood glucose [mg/dl]/2); BMI was calculated using the standard formula: Body mass index = weight / height squared (kg/m2). BMI was categorized according to standard classifications; TyG-BMI Index: TyG-BMI Index = TyG Index*BMI (17).

### Other covariables

The selection of covariates was guided by previous research (22–24). Laboratory tests include determination of triglycerides and glucose. Trained interviewers collected sociodemographic and health-related data through structured questionnaires, including age, sex, place of residence, height, weight, and education level (classified as illiterate primary school, middle school high and above). Residency status is classified as either North or South. Ethnic groups are classified as Han Chinese and other ethnic groups. Health-related variables include self-reported smoking (classified as never, before, or present) and alcohol consumption status (classified as never, now), self-reported physician-diagnosed medical conditions (diabetes, hypertension, stroke, and dyslipidemia).

### Statistical analysis

Data analysis was performed using R software (version 4.2.2). The following statistical methods were applied: Descriptive statistics were calculated for all variables, including means, standard deviations, and proportions. This provided an overview of the sample characteristics and the distribution of key variables. For continuous variables, the mean ± standard deviation (SD) or median (interquartile range, IQR) was utilized to represent the data, depending on the distribution of the variable. To evaluate the relationships between the TyG index, BMI, TyG-BMI index, and hearing loss, multivariate logistic regression models were employed. These models adjusted for potential confounders such as age, sex, socioeconomic status, and lifestyle factors. The primary outcome was the presence of hearing loss, modeled as a binary dependent variable. To account for potential non-linear relationships between the TyG index, BMI, TyG-BMI index, and hearing loss, restricted cubic splines were utilized. This approach allowed for flexible modeling of complex associations. Subgroup analyses were performed to examine whether the relationships between the indices and hearing loss differed by demographic and health characteristics, such as age groups, gender, and the presence of chronic diseases. This helped identify any variations in the effects across different subpopulations. We used ROC curves to compare the strengths and weaknesses of each model, and DeLong test was used to compare the differences between AUCs. Because the TyG-BMI index is a composite index, in order to assess whether the BMI index is the main driver of the TyG-BMI index, and to check for potential redundancy or collinearity, we calculated the variance inflation factor. We used the “car” package in R to calculate the variance inflation factor of TyG-BMI in each of the three models.

## Results

### Results of variance inflation factors

we calculate the variance inflation factor. Here are the results:In crude model:TyG_BMIQ = Q2: 1.378870, TyG_BMIQ = Q3:1.344899, TyG_BMIQ = Q4: 1.345494. In Model 1: TyG_BMIQ = Q2: 1.395334, TyG_BMIQ = Q3: 1.394111, TyG_BMIQ = Q4: 1.432182. In model2:TyG_BMIQ = Q2: 1.399354, TyG_BMIQ = Q3: 1.409151, TyG_BMIQ = Q4: 1.453618. In model3:TyG_BMIQ = Q2: 1.404636, TyG_BMIQ = Q3: 1.444462, TyG_BMIQ = Q4: 1.600408.

### Results of ROC

But based on my result, the TyG index is not significant, the significance of the TyG-BMI index could be entirely driven by BMI. The combination TyG-BMI (TyG × BMI) might simply reflect BMI’s effect without adding meaningful new information. In order to solve this question, we calculated the AUC values of the three indices. The AUC value of TyG index was 0.5127, the AUC value of BMI index was 0.5574, and the AUC value of TyG-BMI index was 0.5512.

We focused on comparing the differences between the BMI index and the TyG-BMI, with the fortunate two having a significant difference. Here are the results: ROC-BMI and ROC-TyG-BMI, *Z* = 2.0669, *p*-value = 0.03874, AUC of roc-BMI:0.5573919, AUC of roc-TyG-BMI:0.5512046.

### Baseline characteristics of the participants

In this cross-sectional study involving a total of 16,157 participants, we analyzed various health metrics and demographic variables to assess differences between groups based on binary categorical variables. Significant differences were found in BMI, TyG-BMI, sex distribution, educational attainment, geographic region, hypertension, diabetes, stroke prevalence, and smoking status between the two groups. No significant differences were observed in glucose levels, triglycerides, TyG index, dyslipidemia, drinking status, or race (Table 1).

**Table 1:** Baseline characteristics of the participants.

Variable	Total (*n* = 16,157)	No (*n* = 14,797)	Yes (*n* = 1,360)	*p*-value
glucose_mg.dL	105.07 ± 32.61	105.07 ± 32.46	105.11 ± 34.23	0.97
TG_mg.dL	134.76 ± 98.16	135.02 ± 97.92	131.97 ± 100.69	0.28
BMI	23.75 ± 3.82	23.81 ± 3.81	23.09 ± 3.85	**<0.0001**
TyG	8.67 ± 0.64	8.67 ± 0.64	8.65 ± 0.64	0.21
TyG-BMI	206.66 ± 40.91	207.22 ± 40.86	200.48 ± 40.92	**<0.0001**
**Sex**				**0.04**
Female	8,731 (54.04)	8,033 (54.29)	698 (51.32)	
Male	7,426 (45.96)	6,764 (45.71)	662 (48.68)	
**Education**				**<0.0001**
Illiterate	1,415 (8.76)	1,350 (9.12)	65 (4.78)	
Primary school	7,328 (45.35)	6,529 (44.12)	799 (58.75)	
Middle school	3,704 (22.93)	3,497 (23.63)	207 (15.22)	
High and above	3,710 (22.96)	3,421 (23.12)	289 (21.25)	
**Location**				0.36
North	7,418 (45.91)	6,777 (45.80)	641 (47.13)	
South	8,739 (54.09)	8,020 (54.20)	719 (52.87)	
**Race**				0.20
Han	15,052 (93.16)	13,773 (93.08)	1,279 (94.04)	
Others	1,105 (6.84)	1,024 (6.92)	81 (5.96)	
**Hypertension**				**<0.0001**
No	11,471 (71.00)	10,579 (71.49)	892 (65.59)	
Yes	4,686 (29.00)	4,218 (28.51)	468 (34.41)	
**Dyslipidemia**				0.83
No	14,053 (86.98)	12,867 (86.96)	1,186 (87.21)	
Yes	2,104 (13.02)	1,930 (13.04)	174 (12.79)	
**Diabetes**				**0.03**
No	14,892 (92.17)	13,660 (92.32)	1,232 (90.59)	
Yes	1,265 (7.83)	1,137 (7.68)	128 (9.41)	
**Stroke**				**<0.001**
No	15,766 (97.58)	14,458 (97.71)	1,308 (96.18)	
Yes	391 (2.42)	339 (2.29)	52 (3.82)	
**Smoke**				**<0.0001**
Former smoker	1,751 (10.84)	1,552 (10.49)	199 (14.63)	
Never smoke	9,806 (60.69)	9,038 (61.08)	768 (56.47)	
Still smoke	4,600 (28.47)	4,207 (28.43)	393 (28.90)	
**Drink**				0.12
No	9,085 (56.23)	8,348 (56.42)	737 (54.19)	
Yes	7,072 (43.77)	6,449 (43.58)	623 (45.81)	
**BMI**				**<0.0001**
Normal	10,684 (66.13)	9,692 (65.50)	992 (72.94)	
Obese	839 (5.19)	786 (5.31)	53 (3.90)	
Overweight	4,634 (28.68)	4,319 (29.19)	315 (23.16)	

### Associations between the TyG index, BMI index, TyG-BMI index and hearing loss

In Table 2, Regarding BMIQ, a significant association with hearing problems was observed in Q4 of the crude model (OR 1.63, 95% CI 1.39, 1.91, *p* < 0.0001) and in Q4 of Model 1 (OR 1.49, 95% CI 1.26, 1.76, *p* < 0.0001). These associations remained significant after adjustments in Models 2 and 3 (Q4: OR 1.54, 95% CI 1.30, 1.82, *p* < 0.0001 in Model 2; OR 1.73, 95% CI 1.45, 2.05, *p* < 0.0001 in Model 3). The trend tests showed significant results across quartiles (p for trend: <0.0001, <0.0001, <0.0001, and <0.0001). For the combined TyG-BMIQ measure, significant associations were noted in the higher quartiles of Q3 and Q4, with increasing ORs (Q2: OR 1.18, 95% CI 1.02, 1.37, *p* = 0.03; Q3: OR 1.5, 95% CI 1.28–1.75, *p* < 0.0001; Q4: OR 1.49, 95% CI 1.27–1.74, *p* < 0.0001). These findings persisted in adjusted models, showing significant trends (p for trend: <0.0001 in crude model; <0.0001 in Model 1; <0.0001 in Model 2; <0.0001 in Model 3).

**Table 2:** Relationship between the TyG index, BMI index, TyG_BMI index and hearing loss.

Character	Crude model		Model 1		Model 2		Model 3	
	95%CI	*p*	95%CI	*p*	95%CI	*p*	95%CI	*p*
TyGQ								
Q1	ref		ref		ref		ref	
Q2	1.03 (0.89, 1.21)	0.67	1.01 (0.86, 1.18)	0.89	1.01 (0.87, 1.18)	0.87	1.03 (0.88, 1.20)	0.72
Q3	1.05 (0.90, 1.22)	0.57	1.01 (0.86, 1.18)	0.94	1.01 (0.87, 1.19)	0.87	1.05 (0.89, 1.23)	0.57
Q4	1.14 (0.97, 1.34)	0.10	1.07 (0.91, 1.26)	0.39	1.08 (0.92, 1.27)	0.34	1.16 (0.98, 1.37)	0.08
p for trend		0.11		0.43		0.37		0.09
BMIQ								
Q1	ref		ref		ref		ref	
Q2	1.17 (1.01, 1.36)	0.04	1.12 (0.96, 1.30)	0.14	1.13 (0.98, 1.32)	0.10	1.16 (1.00, 1.35)	0.05
Q3	1.41 (1.21, 1.65)	<0.0001	1.29 (1.10, 1.51)	0.001	1.32 (1.13, 1.55)	<0.001	1.41 (1.20, 1.65)	<0.0001
Q4	1.63 (1.39, 1.91)	<0.0001	1.49 (1.26, 1.76)	<0.0001	1.54 (1.30, 1.82)	<0.0001	1.73 (1.45, 2.05)	<0.0001
p for trend		<0.0001		<0.0001		<0.0001		<0.0001
TyG_BMIQ								
Q1	ref		ref		ref		ref	
Q2	1.18 (1.02, 1.37)	0.03	1.12 (0.97, 1.31)	0.13	1.14 (0.98, 1.32)	0.10	1.16 (1.00, 1.35)	0.05
Q3	1.5 (1.28, 1.75)	<0.0001	1.38 (1.18, 1.62)	<0.0001	1.41 (1.20, 1.66)	<0.0001	1.51 (1.28, 1.77)	<0.0001
Q4	1.49 (1.27, 1.74)	<0.0001	1.36 (1.15, 1.60)	<0.001	1.4 (1.19, 1.64)	<0.0001	1.58 (1.33, 1.87)	<0.0001
p for trend		<0.0001		<0.0001		<0.0001		<0.0001

The data are presented as odds ratios (ORs), 95% confidence intervals (CIs), and *p* values.

Crude model: unadjusted.

Model 1: adjusted for age, sex, education, location, race.

Model 2: adjusted for Model 1, smoking, drink.

Model 3: adjusted for Model 2, hypertension, dyslipidemia, diabetes, stroke.

### Associations between the TyG index, BMI index, TyG-BMI index and hearing loss

**Figure 2:**
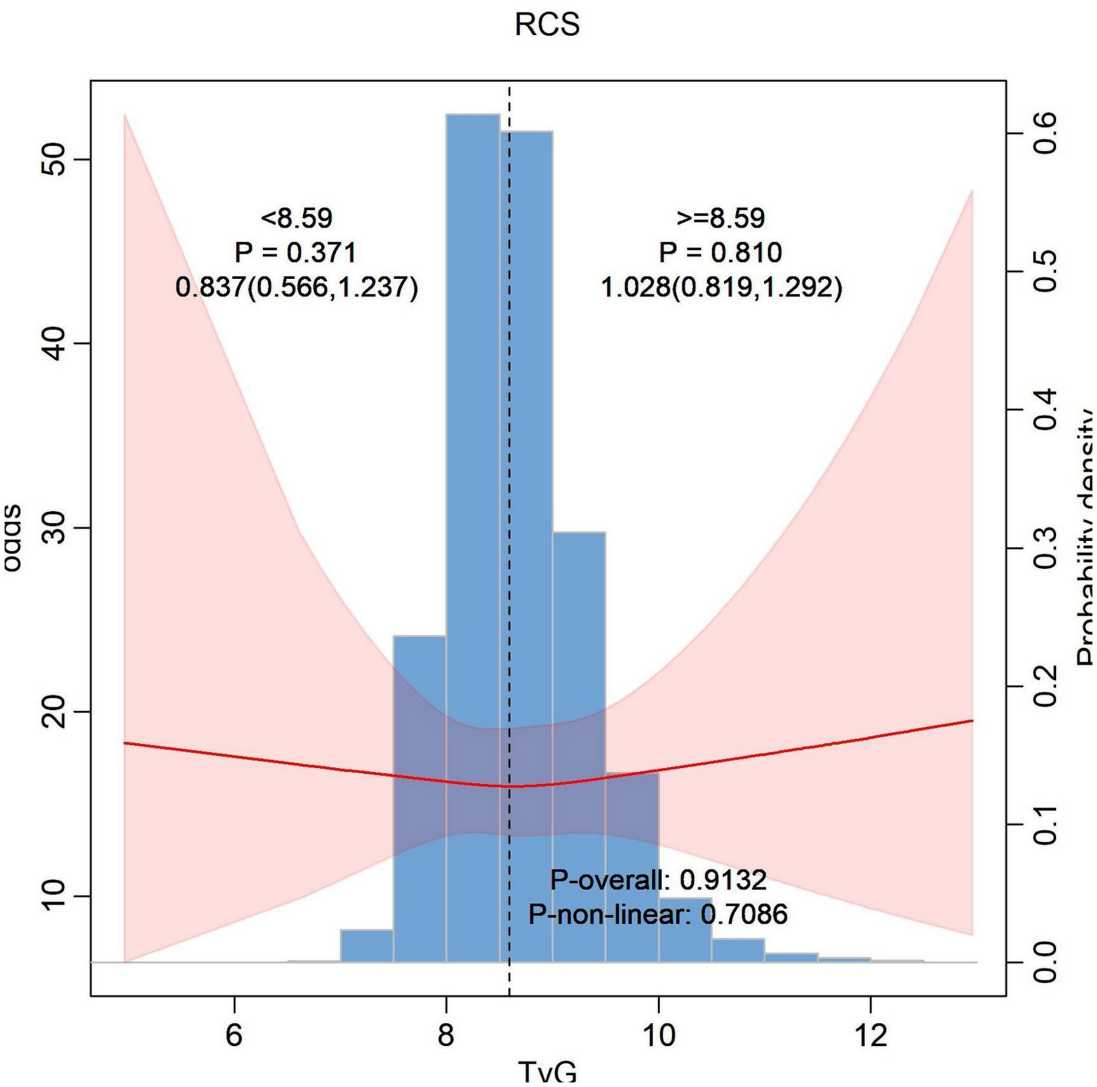
Non-linear relationship between hearing loss and TyG index.

**Figure 3:**
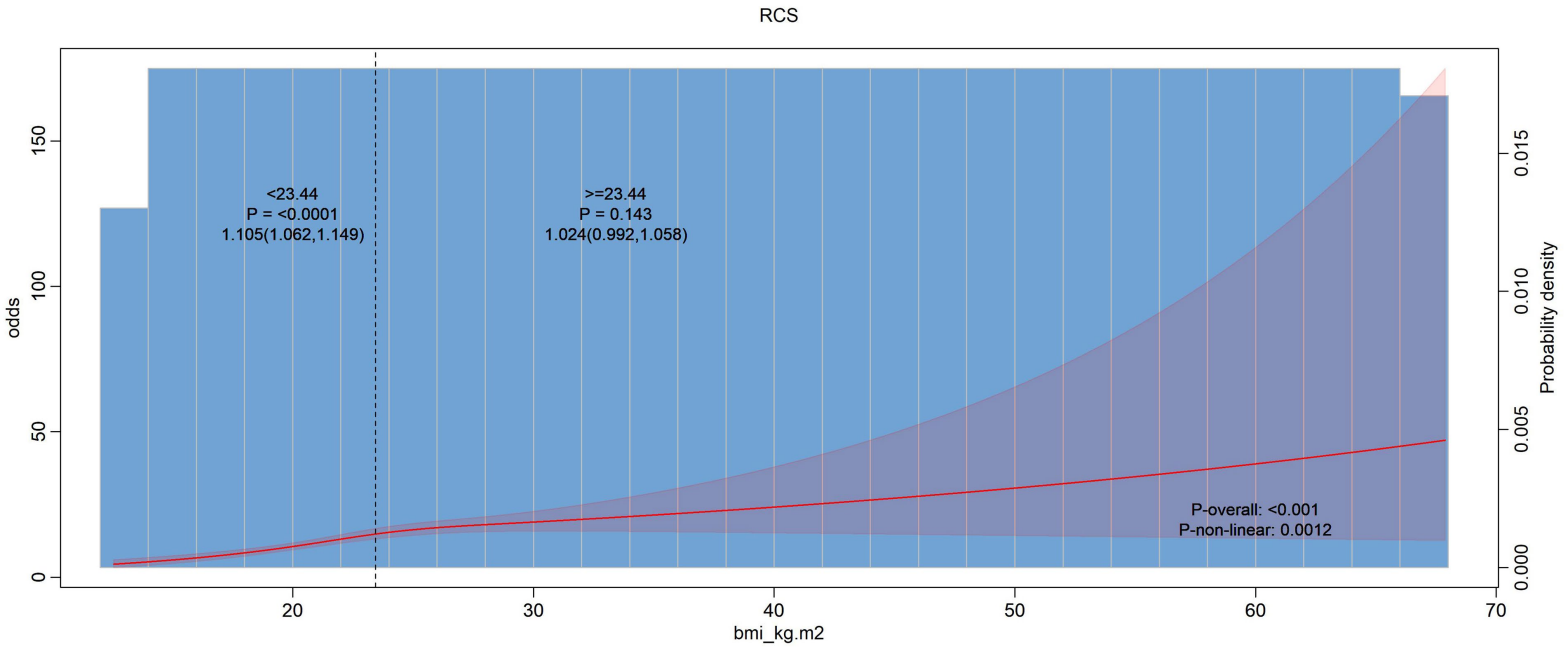
Non-linear relationship between hearing loss and BMI index.

**Figure 4:**
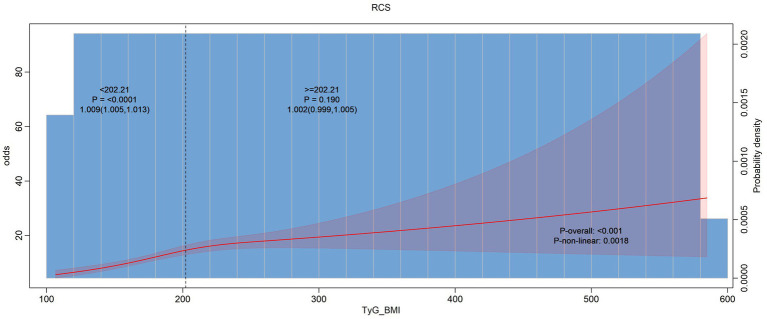
Non-linear relationship between hearing loss and TyG-BMI index.

In Figure 2 our analysis suggests that the TyG index whether considered as a continuous variable or in piecewise segments, does not have a significant association with hearing problems. But in Figure 3 the overall association between BMI and hearing problems was statistically significant. This indicates that a higher BMI is associated with a increased likelihood of having hearing problems. While the standard model indicates a significant association between BMI and hearing problems, the piecewise model does not show significant variations in this relationship across different BMI ranges. Same as Figure 3 in Figure 4 we assessed the association between the TyG-BMI index and hearing problems using both standard and piecewise models. The standard model shows a statistically significant association between the TyG-BMI index and hearing problems, suggesting a slight increase in the likelihood of hearing problems with an increase in the TyG-BMI index.

### Subgroup analysis to the TyG index, BMI index, TyG-BMI index and hearing loss

**Figure 5:**
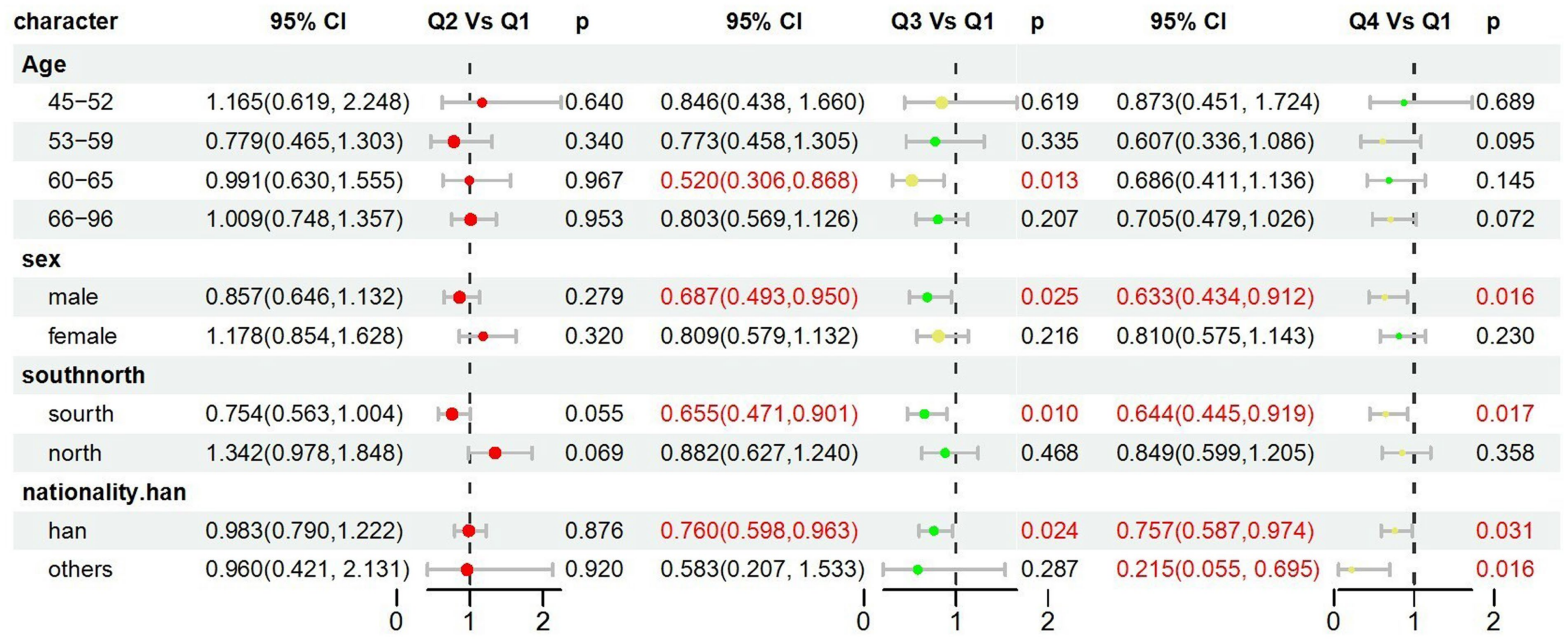
Subgroup analysis to the TyG index, BMI index, TyG-BMI index and hearing loss.

We performed subgroup analysis to stratify the relationship between the change in the TyG-BMI index and hearing problems as shown in Figure 5. We observed significant associations with the outcome were observed in different demographic subgroups, particularly in age groups 60–65 years and 66–96 years, males, southern regions, and Han individuals.

## Discussion

### Overview of findings

This study aimed to explore the relationship between the TyG-BMI index and hearing loss, utilizing data from the CHARLS database. Through a combination of multi-variable logistic regression, linear regression, restricted cubic splines, and subgroup analyses, we investigated how the TyG index, BMI, and TyG-BMI index correlate with hearing impairment. Our results indicate that while the TyG index alone does not show a significant association with hearing loss, both BMI and the TyG-BMI index demonstrate a positive correlation with hearing impairment.

This study explores the relationship between the TyG index, BMI, and the TyG-BMI index with hearing loss, particularly in the context of aging and public health. Hearing loss is a growing concern as global populations age, posing challenges not only for individuals but also for healthcare systems. As hearing impairment affects communication, social engagement, and quality of life, identifying modifiable risk factors like BMI and TyG-BMI is crucial.

While the TyG index did not show a significant association with hearing loss, both BMI and TyG-BMI indices exhibited a positive correlation, particularly in older adults. This suggests that individuals with higher BMI or TyG-BMI may face increased risk of hearing loss with age, highlighting the need for targeted interventions in aging populations.

### TyG index and hearing loss

The TyG index, which combines triglycerides and glucose levels to estimate insulin resistance, did not exhibit a significant relationship with hearing loss in our study. This finding is somewhat unexpected, given that insulin resistance is commonly linked to various health issues, including cardiovascular diseases and metabolic syndrome. This study produced the opposite results from a previous study. Liu’s study suggested that participants with a higher TyG index may have a higher risk of hearing loss (25). There is a linear relationship between the TyG index and HI risk. Pan’s findings suggested that the TyG index had a nearly U-shaped association with speech-frequency and high-frequency hearing thresholds (26).

One potential explanation for the lack of a significant association could be that the TyG index, while useful for assessing metabolic disturbances, may not fully capture the complex interactions between metabolic and auditory health. Hearing loss could be influenced by a range of metabolic factors not solely reflected in the TyG index (25). Another possible explanation is that this study focuses on a different age group than other studies, which focus on people over 45 years of age, while other studies have a larger age span. Second, this study is mainly based on Chinese, and there may be racial differences. Another consideration is the potential measurement limitations (27). The CHARLS database, being cross-sectional, provides a snapshot rather than a longitudinal perspective. As such, it might miss temporal changes or developments in metabolic conditions that affect hearing over time. Longitudinal studies could offer deeper insights into how changes in insulin resistance, as indicated by the TyG index, might correlate with hearing loss over an extended period.

### BMI and hearing loss

Our study found a significant positive correlation between BMI and hearing loss. This finding aligns with existing literature that suggests obesity is a risk factor for sensory impairments. Higher BMI is often associated with systemic inflammation, increased oxidative stress, and changes in blood flow, all of which could contribute to auditory dysfunction. Obesity-related inflammation can affect the inner ear and auditory pathways, potentially leading to deterioration in hearing function (28–30).

Additionally, elevated BMI is commonly linked with other health conditions such as hypertension and diabetes, which are known to impact hearing health. These interconnected factors could exacerbate the risk of hearing loss, providing a plausible explanation for the observed association (31–33).

### TyG-BMI index and hearing loss

More and more studies have shown that insulin resistance and obesity are risk factors for tinnitus and hearing loss. Insulin resistance-induced microangiopathy and peripheral neuropathy lead to hearing loss due to inadequate blood supply to the inner ear (34, 35). while capillary wall tension caused by obesity may be responsible for damage to the delicate inner ear system (30, 36, 37).

Previous studies have shown that IR is strongly associated with hearing loss. Therefore, the TyG index was developed to assess IR (insulin resistant) (34, 35). In addition, BMI is an indicator of obesity and IR. Then, studies of the Chinese population showed that TyG-BMI was strongly associated with HOMA-IR in non-diabetic patients (34). The Korea National Health and Nutrition Examination Survey showed that the TyG-BMI index was superior to other parameters of IR as assessed by HOMA-IR (38).

The most notable finding of this study is the positive correlation between the TyG-BMI index and hearing loss. The TyG-BMI index, which integrates both insulin resistance and body fat metrics (39, 40), appears to offer a more comprehensive view of metabolic health and its impact on auditory function. By combining these two dimensions, the TyG-BMI index may better reflect the complex relationship between metabolic disturbances and hearing impairment.

The TyG-BMI index could capture the cumulative effects of metabolic syndrome and obesity on hearing health, which might be missed when examining TyG or BMI in isolation (41, 42). The positive correlation observed suggests that individuals with higher TyG-BMI scores, indicative of both significant insulin resistance and higher BMI, are at greater risk of experiencing hearing loss. This finding underscores the importance of considering both metabolic and adiposity factors in understanding auditory health. BMI alone ignores body fat distribution, and BMI only considers the ratio of weight to height, and cannot distinguish the ratio of fat to muscle. For example, a muscular athlete may be incorrectly classified as overweight or obese when not truly metabolically risky. At the same time, the BMI index does not reflect insulin resistance, and the BMI itself does not take into account factors such as insulin resistance or metabolic syndrome. Not all obese people may have metabolic problems, while some non-obese people may have insulin resistance, and BMI does not provide details on this. However, the normal range of BMI may be different for different races, genders, and age groups, and some groups of people may be more likely to develop abdominal obesity, which limits the interpretation and applicability of BMI. Ultimately, although BMI is associated with some diseases (e.g., cardiovascular disease, diabetes), it is not as directly associated with some metabolic diseases as the TyG index. Therefore, relying solely on BMI to assess health risk may be incorrect. The TyG-BMI is a combination of the TyG index (a measure closely related to insulin resistance) and the BMI (body mass index) to provide a more complete picture of metabolic health. BMI primarily reflects the ratio of weight to height, while the TyG index reflects insulin resistance, so the TyG-BMI may better capture health problems associated with metabolic diseases. Association with insulin resistance: The TyG index performs well in reflecting insulin resistance, diabetes risk, and metabolic disorders. Combining TyG with BMI may help to more accurately assess the risk of metabolic syndrome and other metabolic diseases. Combining the information from both, the TyG-BMI may be more effective in predicting the risk of cardiovascular disease, diabetes, hearing loss, and other diseases in some cases than BMI or TyG alone. At the same time, the variance inflation factor < 10. This is generally considered an “acceptable” range. Although there is some correlation between them, it is not enough to have a serious impact on regression analysis, so it can be said that there is no significant multicollinearity between these two variables.

From a public health perspective, these findings underscore the importance of age-inclusive approaches in hearing loss prevention. Promoting healthier lifestyles, including weight management and metabolic health, could reduce hearing loss burden in older adults. Furthermore, raising awareness about the importance of early hearing health and regular check-ups across all ages could help prevent or delay hearing impairment.

Future research should investigate the biological mechanisms linking these indices to hearing loss, particularly considering age-related changes in metabolism and vascular health. Understanding these mechanisms will enable the development of more targeted, age-specific interventions.

## Conclusion

This study reveals that both BMI and TyG-BMI indices are associated with an increased risk of hearing loss, particularly in older adults. Although the TyG index alone did not show a significant correlation, the positive association between BMI and TyG-BMI with hearing impairment emphasizes the importance of metabolic health and weight management in mitigating hearing loss. As the global population ages, hearing loss becomes an escalating public health challenge that requires immediate attention.

From a public health perspective, it is crucial to adopt a more inclusive approach that addresses hearing health across the lifespan. While older adults are particularly vulnerable, preventive strategies should also target younger populations. Early interventions aimed at promoting healthy weight, metabolic health, and overall well-being could significantly delay or prevent the onset of hearing impairment in later life.

Furthermore, integrating hearing loss prevention into broader public health campaigns is essential. These should not only focus on the older adult but also emphasize the importance of maintaining hearing health from early adulthood onward. Preventive measures such as regular hearing check-ups, health education, and promoting healthy lifestyle choices, including weight management and metabolic control, can substantially reduce the long-term burden of hearing loss.

Future research should delve deeper into the biological mechanisms that link metabolic factors, such as BMI and TyG-BMI, to hearing function, particularly with aging. Understanding these pathways will inform more targeted and age-specific interventions to prevent or delay hearing loss across diverse age groups.

In conclusion, while BMI and TyG-BMI are significant risk factors for hearing loss, especially in older adults, a comprehensive public health approach is needed. This should include preventive strategies that span the entire lifespan, addressing both metabolic health and hearing function to reduce the societal burden of hearing loss in aging populations while promoting overall well-being at all ages.

### Limitations

This study, while offering valuable insights, is not without limitations. The cross-sectional nature of the CHARLS database restricts our ability to infer causality. Furthermore, self-reported data and potential measurement errors could introduce bias. Future research should address these limitations by employing longitudinal studies and objective measures of both metabolic health and hearing function. In addition, there are numerous factors that can influence hearing, including but not limited to environmental noise levels, exposure to external physical forces (such as loud sounds or trauma), aging, genetic predispositions, lifestyle choices, and underlying health conditions. For instance, prolonged exposure to high levels of noise in one’s living or working environment can contribute significantly to hearing loss over time. Similarly, factors like ear infections, ototoxic medications, and other medical conditions can also play a crucial role in deteriorating auditory function. However, due to the inherent limitations of the CHARLS (China Health and Retirement Longitudinal Study) database, it is challenging to incorporate all these potential contributing factors into the analysis. The database primarily focuses on demographic and socioeconomic data, health conditions, and basic lifestyle factors, but lacks detailed information on some specific auditory-related risk factors, such as the extent of environmental noise exposure or specific medical history related to hearing. As a result, the study’s ability to fully capture and account for all the variables influencing hearing is limited. This is one of the key limitations of the current study. At the same time, we can collect clinical data based on this study, hoping to make our next research closer to the real clinical practice.

## Data Availability

The data presented in the study are deposited in the CHARLS Database repository, This data can be found at: https://charls.pku.edu.cn/.
